# East Tennessee Dental Association

**Published:** 1869-11

**Authors:** 


### Proceedings of Societies.

### EAST TENNESSEE DENTAL ASSOCIATION.

The East Tennessee Dental Association met pursuant to adjournment, at the Lamar House, Knoxville, Tenn., Oct. 20, 1869, at 3 P. M.

The Association was called to order by the President, Dr. John Fouche, who ordered the minutes of the last meeting to be read, after which several new members were elected. The increase in members, both upon the rolls and of the members present, show the growing interest manifested in this body and its objects. An interesting paper on Hemorrhage after Extraction, was read by Dr. Wm. F. Fowler—the various remedies and means for arresting the same, were discussed, after which the Association adjourned to meet next morning at 8 o'clock.

Second Day.—The morning was occupied by a very interesting and instructive clinic by Dr. J. T. Cazier.

The rest of the day was consumed by discussions upon the various methods of treating superficial decay, removing salvary calculus, etc. Also, the different modes of filling teeth.

Third Day.—A clinic also was held by Dr. John Fouche, in the forenoon which was witnessed also with the greatest interest—the Association feeling greatly benefited by the clinics of the two mornings.

Two essays were read by Dr. Wm. H. Cook, one on the Necrosis of the Alveoli, the other on Tumors of the Mouth. Both were highly entertaining and instructive.

Dr. Cazier drew up, and presented to the Association a bill memorializing the Legislature of the State of Tennessee, to pass the same for the better regulating the practice of Dentistry in Tennessee.

Drs. Fouche, Cazier and Cooke were elected to represent the Association at the next meeting of the American Dental Association, which convenes in the city of Nashville. Also, Drs. Fowler and Speck were elected to represent the interests of the same, at the next meeting of the Southern Dental Association at New Orleans.

Several members having to leave, the Association adjourned after electing the following officers for the ensuing year, to-wit:

*President*, Dr. J. T. Cazier; *Cor. Secretary*, Wm. F. Fowler; *Rec. Sec. and Treasurer*, Wm. H. Cooke.

